# Metformin promotes apoptosis in primary breast cancer cells by downregulation of cyclin D1 and upregulation of P53 through an AMPK-alpha independent mechanism

**DOI:** 10.3906/sag-1908-112

**Published:** 2021-04-30

**Authors:** Güven YENMİŞ, Nail Beşli, Elif YAPRAK SARAÇ, Fatma Sinem HOCAOĞLU EMRE, Kazım ŞENOL, Gönül KANIGÜR SULTUYBEK

**Affiliations:** 1 Department of Medical Biology, Faculty of Medicine, Biruni University, İstanbul Turkey; 2 Department of Medical Biology, Faculty of Medicine, Sağlık Bilimleri University, İstanbul Turkey; 3 Department of Molecular Biology and Genetics, Faculty of Science and Letters, İstanbul Technical University, İstanbul Turkey; 4 Department of Nutrition and Dietetics, Faculty of Health Sciences, Beykent University, İstanbul Turkey; 5 Department of General Surgery, Faculty of Medicine, Bursa Uludağ University, Bursa Turkey; 6 Department of Molecular Biology and Genetics, Faculty of Medicine, Istanbul Aydın University, İstanbul Turkey

**Keywords:** AMPK-alpha, cyclin D1, Tp53, breast cancer, metformin

## Abstract

**Background/aim:**

In the present study we aimed to figure out the effect of metformin on the expression of AMPK-alpha, cyclin D1, and Tp53, and apoptosis in primary breast cancer cells (PBCCs).

**Materials and methods:**

PBCCs were treated with two doses of metformin (0 mM, 25 mM). Proliferation was determined by BrdU assay. Real-time PCR was used to assess AMPK-alpha, cyclin D1, and Tp53 gene expressions; apoptotic indexes of PBCCs were analyzed using flow-cytometry.

**Results:**

Twenty-four–hour incubation with 25 mM metformin reduced the proliferation of PBCCs. AMPK-alpha gene expression in PBCCs was not affected by 25 mM metformin treatment compared with the control group. PBCCs treated with 25 mM metformin had lower cyclin D1 expression compared with nontreated cells; however, the difference was not statistically significant. Twenty-five millimolar dose of metformin increased p53 expression significantly compared with the nontreated group. The high concentration of metformin elevated the number of annexin V-positive apoptotic cells, and the increase in the apoptotic index was statistically significant.

**Conclusion:**

Metformin can modulate cyclin D1 and p53 expression through AMPK-alpha-independent mechanism in breast cancer cells, leading to cell proliferation inhibition and apoptosis induction.

## 1. Introduction

Metformin has been the most widely prescribed antihyperglycemic biguanide for treating individuals with type 2 diabetes (T2DM) for decades. It performs its glucose-lowering effect through elevated glucose use and diminished hepatic glucose production [1–3]. In the last decade, a decrease in AMP/ATP ratio was reported to induce indirectly a ubiquitously expressed enzyme—a cellular energy sensor, namely, “AMP-activated protein kinase (AMPK)”[4,5]. 

Metformin, nowadays, is one of the most famous anticancer drugs used in cancer treatment. The anticancer effect of metformin is considered to be partial via inhibition of cell proliferation [6–8]. Through downregulation of cyclin D1 expression and upregulation of p21, metformin, in breast, bladder, and prostate cancer cells, blocks the cell cycle in the G1 phase [9–11]. Besides, the upregulation of AMPK has been found in human breast cancer and suggested to be not only a prognostic factor but also a therapeutic target [12]. 

There is a general agreement that p53 is involved not only in cell metabolism but also in cell cycle control and indirectly in apoptosis and the regulation of both p53 phosphorylation and expression and is regulated by AMPK; its participation, thus, in metformin action is inevitably argued. Interestingly, accumulating through elevation pointed out that metformin without any changes in p53 status arrested cell cycle in the G0/G1” stage with a remarkable reduction of G1 cyclin expression, namely cyclin D1 [13–15]. On the other hand, p53 activity was reported to be related to metformin’s inhibitory effect on cancer cell growth [16–19]. In another valuable research about metformin’s effect on hepatocytes, Yi et al. pinpointed that while a low concentration of metformin stimulates p53-dependent senescence, a high concentration, instead, stimulates apoptotic cell death [20]. Therefore, more association studies of metformin with p53 activity would be highly desired. In the case of breast cancer progression, breast tissue cells transform into malignant ones through the dysregulation of cellular processes such as cell cycle, angiogenesis, and apoptosis [21]. Thus, to combat cancer, up-to-date treatment strategies aim at targeting these processes, more specifically apoptosis [22].

Thus, in the present study, we hypothesized that metformin could block cell cycle progression and induce cell apoptosis through the expression of AMPKα in human PBCCs and the underlying mechanism may involve the dysregulation of cyclin D1 and p53 expression.

## 2. Materials and methods

### 2.1. Cell culture and dosing

Human PBCCs were obtained from biopsies of breast tumors of five human donors in the age range of 45–55 years, who applied to the Department of General Surgery. The selection criteria were being in the postmenopausal period, as well as the positivity of both estrogen and progesterone receptors (Table 1). All study subjects were provided signed informed consent prior to the sample collection. The protocol for establishing primary human cell cultures from biopsies obtained during mastectomy was approved by “the Ethics Committee of Clinical Research Center of Cerrahpaşa School of Medicine, İstanbul University, İstanbul, Turkey (Date: 15th July 2016, Approval number: 83045809 - 604.01.02 - 257133).

**Table 1:** The clinical characteristics of the patients with breast cancer.

	Patient I	Patient II	Patient III	Patient IV	Patient V
Age (years)	45	54	53	49	55
Sex	Female	Female	Female	Female	Female
Diagnosis	IDC+ILC	IDC	IDC	IDC+ILC	IDC
Menopausal status	Post	Post	Post	Post	Post
Famillial breat cancer history	Negative	Negative	Negative	Negative	Negative
Pathology of the tumor	Malignant	Malignant	Malignant	Malignant	Malignant
Size of the tumor in radius (cm)	2.1	2.7	2.4	3	2.9
Histological grade	II	II	II	II	II
Patient follow-up	2 years	>2 years	<1 year	1 year	2 years
Breast cancer surgery	Mastectomy	Mastectomy	Mastectomy	Mastectomy	Mastectomy
ER status	Positive	Positive	Positive	Positive	Positive
PR status	Positive	Positive	Positive	Positive	Positive
HER2 status	Negative	Negative	Negative	Negative	Negative
E-cadherin	Positive	Positive	Positive	Positive	Positive
%Ki 67	40	16	58	60	38

IDC: Invasive ductal carcinoma, ILC: Invasive lobular carcinoma

Breast tissues were cut into slices and cultured for 10–14 days as a monolayer in DMEM/F12 medium”(Wisent Bioproducts, Quebec, Canada)” supplemented with 10% fetal bovine serum”(Wisent Bioproducts, Quebec, Canada), 100 U/mL penicillin (Wisent Bioproducts, Quebec, Canada), and 100 µg/mL streptomycin (Wisent Bioproducts, Quebec, Canada) in a 5% CO2 containing 37 °C humidified atmosphere. PBCCs were sorted by the BD FACS Calibur system (BD Biosciences, USA), according to the protocol previously mentioned by Malecki et al. [23]. Monolayers of the cells in flasks were subcultured on each 6th–8th day using trypsin. Five of these primary cultures were included in the assay and all experiments were repeated five times. The number of cells from the 3th–6th passages was calculated as 300,000–450,000 cells/mL. The cells were then subjected to proliferation assay and flow-cytometry or immediately frozen at –20 °C for quantitative real-time polymerase chain reaction (RT-PCR). All concentrations and incubation time intervals were selected from the earlier literature [24,25].

### 2.2. Br-dU proliferation assay 

Analysis with“5-bromo-2’-deoxyuridine (BrdU) (Sigma-Aldrich, Schnelldorf, Germany)” was performed according to the manufacturer’s protocol.”HRP/AEC (ABC) Detection IHC Kit (Abcam, ab93705, USA)”was used for immunocytochemical staining. A mouse monoclonal BrdU Antibody”(Santa Cruz Biotechnology Inc., sc-20045)”was used as the primary antibody”(1:200, overnight).”Aminoethylcarbazole (AEC) (Invitrogen, Calsbad, USA)”was used as the chromogen. BrdU was measured via visual colorimetric staining. BrdU-labeled cells were evaluated by two analysts and the proliferation index was calculated via the proliferation count equation (positively stained cell number (viable)/total cells counted (N) (viable and nonviable cells) ( N > 100 )). The proliferation count equation was applied for all doses. 

### 2.3. Quantitative real-time PCR (qRT-PCR)

The breast cancer cells were incubated in 0 mM and 25 mM metformin (Met), and then collected and washed with PBS. Total RNA isolation was performed with RNA isolation kit”(QIAamp® RNA Blood Mini kit, QIAGEN Pty Ltd, Victoria, Australia)” according to the manufacturer’s protocol instruction. The RNA quantity and quality were determined by a spectrophotometer “(Nanospectrophotometer P300, IMPLEN, Germany)” and 1 µg total RNA was subjected to reverse transcription-PCR using”Qiagen Quantitech Reverse Transcription Kit (QIAGEN Pty Ltd, Victoria, Australia), according to manufacturer’s protocol instruction. cDNAs were then subjected to quantitative real-time PCR analysis using”Qiagen Quantitect SYBR Green PCR Kit (QIAGEN Pty Ltd, Victoria, Australia)”and self-created primer sets for each gene on a real-time PCR device”(Rotor-Gene Q-Qiagen Pty Ltd, Victoria, Australia). The values of threshold cycles (Ct) for *AMPKα1* , *CCND1* , and *Tp53* mRNA transcripts were calculated in relation to the expression of a house-keeping gene, beta-actin (ACTB). For the used primer sets please see Table 2.

**Table 2:** Speciﬁc primer sets for quantitation analysis of AMPKα1, CCND1, and Tp53 genes (and ACTB as the house-keeping gene).

Primer	Sequence	Spanning exons
AMPKα1_sense	tttgagtgctcagaagaggaa	Exon 7
AMPKα1_antisense	gtgtttcagcaaccaagaatg	
CCND1_sense	cattgaacacttcctctccaa	Exon 3
CCND1_ antisense	ggtcacacttgatcactctgg	Exon 4
Tp53_sense	tctacaagcagtcacagcaca	Exon 5
Tp53_ antisense	gtacagtcagagccaacctca	Exon 6-7
ACTB_sense	atctggcaccacaccttcta	Exon 3
ACTB_ antisense	agcctggatagcaacgtaca	Exon 4

### 2.4. Apoptosis assay

Apoptosis was measured using an Annexin V-FITC apoptosis detection kit I (BMS500FI-100, eBioscience, MA USA) and analyzed by flow-cytometry. Stained cells were acquired by using MacsQuant Analyzer 10 and obtained data were analyzed by using MacsQuantity Version 2.8 flow-cytometry screening system. Briefly, primary PBCCs were treated with different concentrations of metformin”(0 mM and 25 mM)” for 24 h and the flow-cytometry procedures according to the manufacturer’s kit. The apoptotic index was calculated by means of all five repeated experiments and given as a percentage.

### 2.5. Statistical analysis 

Experiments repeated three times were statistically analyzed using GraphPad InStat Software (Version 3.06). Results are presented as means ± standard error mean (SEM) and compared by unpaired t-test with Welch correction. P < 0.05 was accepted as statistically significant.

## 3. Results

### 3.1. Antiproliferative effects of metformin in breast cancer cells

**Figure 1:**
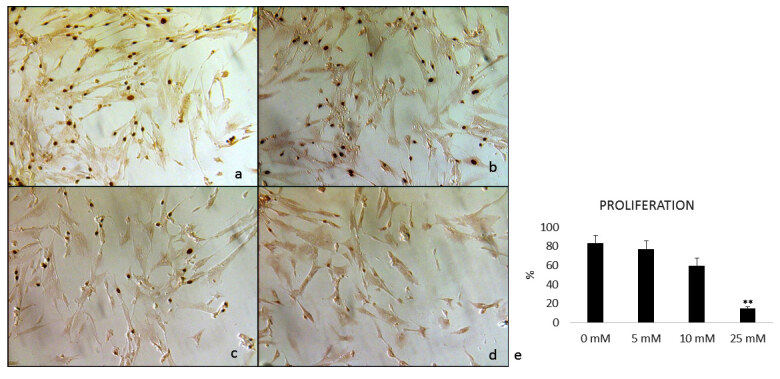
Immunocytochemical proliferation analysis (BrdU) of primary breast cancer cells treated with four concentrations of metformin: a) 0 mM metformin, b) 5 mM metformin, c) 10 mM metformin, d) 25 mM metformin, e) The graph of proliferation indexes, ***P < 0.0001 vs all groups. The number of breast cancer cells significantly decreases in the presence of increasing metformin concentration in a dose-dependent manner.

We determined the proliferation of PBCCs treated with metformin at a concentration of “0, 5, and 10, and 25 mM” for 24 h (Figures 1a–1d). Compared to the untreated (0 mM Met) control cells, metformin inhibited the proliferation of PBCCs in a dose-dependent manner. Statistically, significant inhibition was observed at the 25 mM Met concentration compared to the 0 mM dose, although there was no conformational change in the adherence of the cells upon incubation with any of the doses (P < 0.001) (Figure 1e; Table 3). The metformin concentrations applied in this study are comparatively high, but to concentrate on the anticancer effects of metformin on PBCCs, 25 mM Met was used in the following experiments. All experiments were performed four times in quadruplicate.

**Table 3:** BrdU proliferation indexes of breast cancer cells.%0 mM Met5 mM Met10 mM Met25 mM MetMEAN ± SEM83.48 ± 8.2777.48 ± 8.5859.50 ± 8.3615.30 ± 1.89*

%	0 mM Met	5 mM Met	10 mM Met	25 mM Met
MEAN ± SEM	83.48 ± 8.27	77.48 ± 8.58	59.50 ± 8.36	15.30 ± 1.89*

Met: metformin; SEM: standard error mean; *P < 0.001

### 3.2. Effect of metformin on AMPKα1 expression in breast cancer cultures 

**Figure 2:**
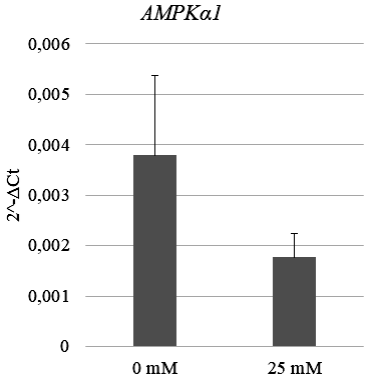
AMPKα1 expression profile of primary breast cancer cells treated with two different concentrations (0 mM and 25 mM) of metformin. The decrease in AMPKα1 expression at 25 mM metformin concentration is not statistically significant in breast cancer cells (P > 0.05).

The expression of the *AMPKα1* gene in the breast cancer cells was examined by qRT-PCR before and after 24-h treatment with metformin. Although the *AMPKα1* expression reduced in the group treated with 25 mM, the change in the expression was not statistically significant (Figure 2), which suggests that the high metformin concentration used in this study did not show either inhibitory or stimulatory effect on *AMPKα* expression compared with the control group (P > 0.05). 

### 3.3. Effects of metformin on cyclin D1 gene (CCND1) expression in breast cancer cultures

**Figure 3:**
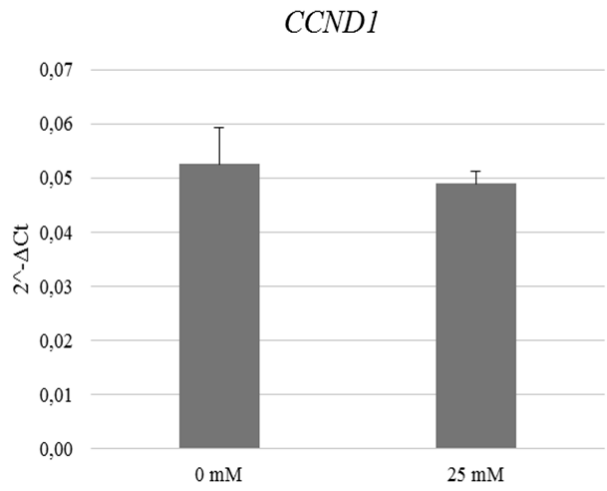
CCND1 expression profile of primary breast cancer cells treated with two different concentrations (0 mM and 25 mM) of metformin. CCND1 expression decreases at 25 mM metformin concentration but the decrease is not statistically significant (P > 0.05).

The expression of the *CCND1* was also detected before and after 24-h treatment of metformin in breast cancer cells, by performing qRT-PCR analysis. According to the results, the *CCND1* expression reduced in the group treated with 25 mM Met compared to the nontreated group but the decrease was not statistically significant (P = 0.64) (Figure 3).

### 3.4. Effects of metformin on Tp53 gene expression in breast cancer cultures

**Figure 4:**
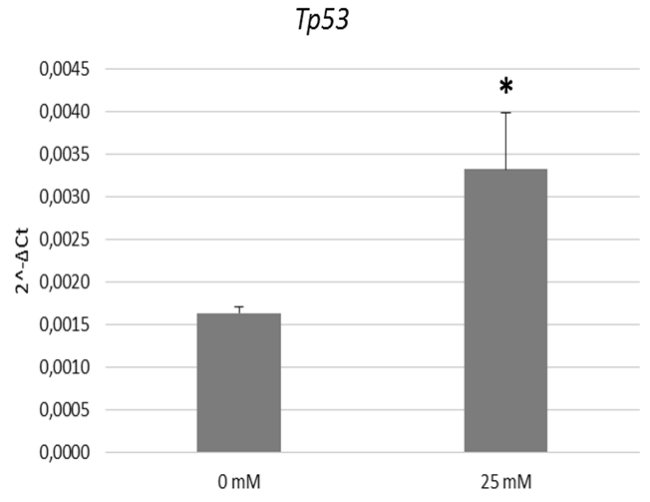
Tp53 expression profile of primary breast cancer cells treated with two different concentrations (0 mM and 25 mM) of metformin (P < 0.05). Tp53 expression is elevated at 25 mM metformin concentration in breast cancer cells.

To quantify the expression of the *Tp53* gene in PBCCs, qRT-PCR was performed. The data revealed that the expression of *Tp53* was significantly elevated by 25 mM Met application compared with 0 mM Met (P < 0.05) (Figure 4), which suggests that metformin selectively has an impact on the expression of the *Tp53* gene. 

### 3.5. Effects of metformin on the apoptosis levels of primary breast cancer cells

**Figure 5:**
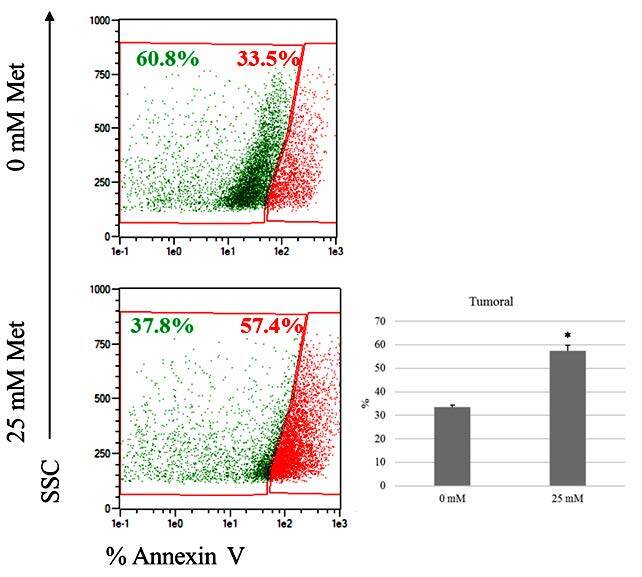
Apoptotic index of primary breast cancer cells treated with two different concentrations (0 mM and 25 mM) of metformin. Apoptosis tendency increased in the presence of 25 mM metformin treatment (P = 0.03).

Figure 5 presents the results for the apoptotic index of PBCCs. To check the high metformin-induced apoptosis, breast cancer cells were treated with two concentrations of metformin for 24 h and followed by annexin V–FITC staining. The high concentration of metformin reduced the number of annexin V positive apoptotic cells, and the increase in the apoptotic index was statistically significant compared with 0 mM metformin-treated cells (P < 0.05).

## 4. Discussion

Nowadays, metformin, as an antitumor agent, gained momentum, leaving metformin-based cancer therapies as an afterthought. Although no conclusive data is present about the function of metformin in both cancer therapy and prevention either in diabetic or in nondiabetic populations, the possible effects are observed indirectly on systemic levels of glucose or insulin, or directly on tumor cell growth via inhibition of cell proliferation and survival via induction of cell death [10,26–30]. Metformin seems to be involved in cancer cell inhibition through AMPK and cyclin D1 downregulation leading to cell cycle arrest and apoptotic pathways activation [9,15,27,31]. AMPK appears to be the hallmark of metformin metabolism in cancer cells. However, the literature has a large controversy in AMPK pathway involvement in cancer progression; until now, no AMPK activation was observed in recent reports of lung, prostate, breast, and colorectal cancer [32]. Conversely, in the current research, we could not find any clues which support the idea that metformin suppresses the proliferation of PBCCs via upregulating AMPKα expression.

Moreover, cyclin D1, a requirement for G1/S transition of the cell cycle [33] and an indirect cell cycle player, p53 [34], was reported to be essential specifically in embryonic fibroblasts of the mouse for AMPK-induced cell cycle arrest [35]. Metformin was reported to stimulate G0/G1 cell cycle arrest, regulated through oxidative stress as well as AMPK activation [36]. In cell cycle analysis studies, S phase cell cycle accumulation with apoptosis enhancement was displayed in triple-negative breast cancer (TNBC) [26] and a pancreatic cancer cell line [37], whereas G1 phase cell cycle accumulation without any apoptosis enhancement was observed in the non-TNBC [10] and an ovarian cancer cell line [38]. Besides, dose-dependent inhibition of proliferation via a reduction in cyclin D1 levels arresting in the G0/G1 stage was observed in gastric [39] and prostate cancer cells [9]. In the present study, we found that cyclin D1 expression of PBCCS is decreased at 25 mM metformin concentration. There is a growing body of evidence suggesting that metformin is able to initiate p53 by both“AMPK-dependent”[8,14] and -independent [17,40] mechanisms to stimulate apoptosis and cell cycle arrest and hence might be essential for cancer therapy. Most importantly, not only the upregulation of AMPKα and p53 but also the downregulation of cyclin D1 are involved in the antitumor action of metformin in vivo. We observed that p53 expression of PBCCS is increased at 25 mM metformin concentration. Thus, all in all, we suggest that metformin induces cell-cycle arrest in PBCCs by downregulating cyclin D1 expression and upregulation of p53 expression through the AMPK-independent pathway.

Nonetheless, in addition to AMPK activation, metformin also induces apoptosis in breast cancer cells independently of AMPK [16]. In the current study, we found that apoptosis of PBCCS is increased at 25 mM metformin concentration correlated with p53 expression and cyclin D1 downregulation. The apparent discrepancy of the presence of AMPK activation in a trace amount of cancers in the presence of metformin uptake may be due to the dissimilarity in used medium, incubation period, and pharmacologic doses. The majority of the studies reviewed utilized DMEM (including 24 mM glucose) or DMEM/F12 (including 17.5 mM glucose) as the culture medium, and the normal plasma glucose concentration was below 7 mM. In this respect, the cytotoxic effect of metformin is proved to be potent in low-glucose media by Zhuang et al., proposing that the requirement of metformin at high concentrations in in vitro experiments is because of the masking effect glucose presents; hence, the data reported in these researches are not invalidated [41].

**Figure 6:**
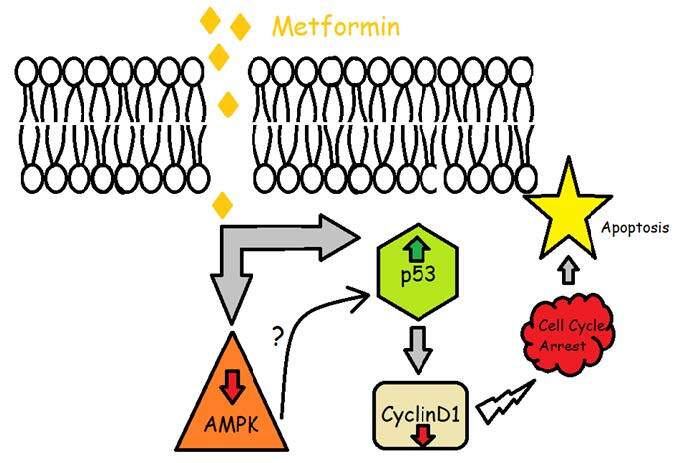
The overall process of the mechanism of metformin on cell cycle arrest and apoptosis. Metformin may regulate apoptosis via two different pathways. Firstly, metformin may decrease AMPK-alpha expression which then indirectly stimulates the cell cycle arrest (AMPK-dependent way). Secondly, metformin may trigger cell cycle arrest in primary breast cancer cells by upregulation of P53 and downregulation of cyclin D1 and which eventually results in cell cycle arrest (AMPK-independent way).

In conclusion, both the inhibition of tumor growth and induction of apoptosis through metformin treatment in breast cancer cells require p53 and cyclin D1 involvement (Figure 6). The current study has some limitations, albeit the promising results. First of all, the expression levels of mRNAs were not confirmed at the protein level. Secondly, the kinase activity of AMPK-a was not determined. Thus, the mechanisms on how metformin displays its inhibitory effects on cancer development and tumor growth are not fully figured out. A couple of milestones yet need to be passed according to this novel antitumor molecular approach. Firstly, the applicability of nondiabetic subjects for the anticancer effect of metformin and the appropriate safety dose should be illuminated. Secondly, in the neoplastic tissue, the adequate drug concentration of metformin should be determined, and thirdly, the benefit of metformin as a clinical marker should be defined in the future.
